# Clinical usefulness of Somatostatin Receptor Scintigraphy in the Diagnosis of Neuroendocrine Neoplasms

**DOI:** 10.22038/AOJNMB.2021.56254.1390

**Published:** 2022

**Authors:** Yoshitaka Inaba, Susumu Hijioka, Isanori Iwama, Tsubasa Asai, Hiroki Miyamura, Shohei Chatani, Takaaki Hasegawa, Shinichi Murata, Mina Kato, Yozo Sato, Hidekazu Yamaura, Hiroaki Onaya, Junichi Shimizu, Kazuo Hara

**Affiliations:** 1 Department of Diagnostic and Interventional Radiology, Aichi Cancer Center Hospital, Nagoya, Aichi, Japan; 2 Department of Gastroenterology, Aichi Cancer Center Hospital, Nagoya, Aichi, Japan; 3 Department of Thoracic Oncology, Aichi Cancer Center Hospital, Nagoya, Aichi, Japan

**Keywords:** Neuroendocrine neoplasms (NEN), Neuroendocrine tumor (NET), Somatostatin receptor scintigraphy (SRS), Indium-111 pentetreotide, Octreo scan

## Abstract

**Objective(s):**

We investigated the detectability of somatostatin receptor scintigraphy (SRS) for neuroendocrine neoplasms (NEN).

**Methods:**

From January 2016 to October 2020, 125 SRS examinations using indium-111 pentetreotide performed for patients with NEN lesions were retrospectively evaluated. The detection rate of NEN lesions was determined according to histopathological classification by primary site and by organ.

**Results:**

At least one NEN lesion was detected in 73% (91/125) with a positive Krenning score of ≥2 in SRS. The detection of abdominal NENs (gastrointestinal tract, 38; pancreas, 62; and others, 14) was 89% (49/55) for neuroendocrine tumor (NET)-grade (G) 1, 78% (32/41) for NET-G2, 66% (2/3) for NET-G3, 31% (4/13) for neuroendocrine carcinoma (NEC), 100% (1/1) for mixed neuroendocrine–non-neuroendocrine neoplasm, and 0% (0/1) for non-classified NEN. That of thoracic NENs was 33% (2/6) for typical carcinoid tumor and 40% (2/5) for atypical carcinoid tumor. For a total of 226 organ lesions, hepatic lesions were 76% (58/76); pancreatic lesions, 61% (31/51); lymph node lesions, 77% (27/35); bone lesions, 83% (20/24); duodenal lesions, 82% (9/11); and other lesions, 41% (11/27).

**Conclusion:**

The detectability of SRS for NEN in Japan was verified at a center, and its usefulness was confirmed.

## Introduction

 Somatostatin receptor scintigraphy (SRS) using indium-111-labeled pentetreotide (In-111 pentetreotide) was launched in January 2016 and became available for the diagnosis of neuroendocrine neoplasm (NEN) in Japan. SRS exhibits a high detection sensitivity and clinical efficiency in patients of gastrointestinal and pancreatic NENs; it has been introduced since the 1990s and has become a standard test method of NEN diagnosis in Europe and the United States (1).

 In-111 pentetreotide is a preparation of pentetreotide in which the chelating agent DTPA (diethylenetriaminepentaacetic acid) is bound to octreotide, which was developed as a 

somatostatin analog (SSA), labeled with radioactive indium-111. Similar to octreotide, it has affinity for somatostatin receptor (SSTR) 2, SSTR3, and SSRT5 among the five subtypes of SSTRs (SSTR 1–5), with a particularly high affinity for SSTR2. SSTR2 and SSTR5 are highly expressed in the cell membrane of NEN, leading to the application of SRS for NEN diagnosis (1, 2).

 Our hospital has been performing SRS examination since January 2016, when the procedure became available in Japan (3). NEN is considered a rare disease. However, considering that the number of patients subjected to SRS examination exceeded 100, the detectability of SRS was compared with computed tomography (CT) and fluorine-18 deoxyglucose positron emission tomography (FDG-PET), and its usefulness was retrospectively verified.

## Methods

***Survey target***

 From radiological diagnostic image and report servers of our hospital, we extracted data of cases that underwent SRS examination. The diagnosis obtained as per the SRS diagnostic report, CT report performed within 3 months before and after the SRS examination, and FDG-PET/CT diagnosis report was compared. Contrast-enhanced CT was typically performed; however, non-enhanced CT was performed in patients in whom contrast medium could not be used. Medical history, examination purpose, presence/absence of treatment with SSA, pathological diagnosis reports created by pathologists at our facility, diagnostic imaging reports created by diagnostic radiologists at our facility, and examination images were obtained from patient medical records. The histopathological classification of NEN was determined based on the Ki-67 index and the number of mitoses in accordance with the World Health Organization (WHO) classifi-cation (4, 5). This retrospective study was approved by the Ethical Review Committee of our hospital (approval no.: 2018-1-089). Written informed consent about this study for all patients was waived, but an opportunity to opt out was provided.

 From January 2016 to October 2020, a total of 145 consecutive SRS examinations were conducted. During the study period, 2, 2, 1, 13, and 98 patients underwent the SRS examination 5, 4, 3, 2, and 1 times, respectively. A total of 116 patients were examined; the interval between the examinations was ≥10 months, and each examination was investigated. The presence or absence of target lesions in SRS examination was determined based on overall assessment, including CT, FDG-PET/CT, gastrointestinal endoscopy, and magnetic resonance imaging (MRI) findings.

**Figure 1:**
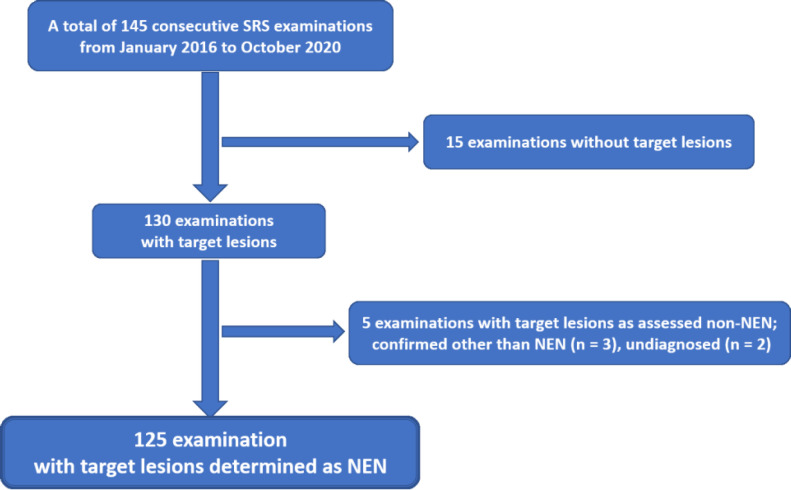
Survey target

 The objective of the SRS examinations was as follows: 15 examinations were performed without target lesions, recurrence after NEN resection was investigated in 14 examinations, and NEN lesion was investigated due to high serum gastrin levels in 1 examination. In 130 examinations, patients had target lesions. The pre-resection evaluation for NEN or suspected NEN lesions was performed in 34 examinations, pre-non-resection treatment evaluation was conducted in 22 examinations, evaluation of chemotherapy was performed in 63 examinations, observation without treatment was the purpose for 7 examinations, and diagnosis was the purpose for 4 examinations. Pathological diagnoses of the evaluated lesions other than NEN after resection were confirmed in 3 examinations (renal cell carcinoma with pancreatic metastasis=1, solid pseudopapillary neoplasm of the pancreas=1, and gangliocytic paraganglioma of the duodenal papilla=1), and 2 examinations (both were suspected as pancreatic NEN) remained undiagnosed despite performing biopsy. Pathological diagnoses of NEN based on biopsy and resected specimens were obtained in 125 examinations (Figure 1). Table 1 shows the patient background in these 125 SRS examinations. The detectability of NEN lesions in these 125 SRS examinations performed on patients with NEN lesions, who were 66 males and 59 females with a median age of 64 years (age range 31-84 years), was evaluated. Gastrointestinal NEN was identified in 38 examinations, pancreatic NEN in 62 examinations, other abdominal NEN in 14 examinations, lung NEN in 9 examinations, and thymic NEN in 2 examinations. They were evaluated as per the histopathological classification of NEN by the primary organ.

**Table 1:** Patient background on 125 SRS examinations with NEN lesions

**Patient background**	**Number of subjects**
SRS examinations, n	125
Sex, n	Male 66, Female 59
Median age, years (range)	64 (31-84)
	
Non-functinal NEN, n (%)	114 (91.2)
Functinal NEN*, n (%)	11 (8.8)
MEN 1 related NEN, n (%)	9 (7.2)
Double primary NEN**, n (%)	7 (5.6)
Objective of SRS examinations, n (%)	
pre-resection evaluation	31
pre-non-resection evaluation	22
chemotherapy evaluation	63
observation without treatment	7
diagnosis	2

SRS: somatostatin receptor scintigraphy

NEN: neuroendocrine neoplasms

MEN 1: Multiple endocrine neoplasia type 1

NET-G: neuroendocrine tumor-grade

*high gastrin levels (n = 12), high vasoactive intestinal polypeptide levels (n = 1)

**These included pancreatic NET-G1 + duodenal NET-G1 (n = 5), pancreatic NET-G2 + rectal NET-G1 (n = 1), and gastric NET-G1 + rectal NET-G2 (n = 1). One primary lesion with high malignant grade or widespread development was designated as the representative primary lesion in this study; duodenal NET-G1 (n = 4), pancreatic NET-G1 (n = 1), and rectal NET-G2 (n = 2).

 The histopathological grade of NEN was classified according to WHO criteria (WHO2019 for gastroenteropancreatic (GEP) NEN (4), WHO2015 for lung NEN (5)) based on the differentiation, Ki-67 index, and the number of mitoses per 10 high power fields (HPF), the degree of necrosis. GEP NEN was classified as neuroendocrine tumor (NET) grade (G) 1 (well-differentiated, Ki-67 <3%, <2 mitoses/10 HPF), NET-G2 (well-differentiated, Ki-67 3-20%, 2-20 mitoses/10 HPF), NET-G3 (well-differentiated, Ki-67 >20%, >20 mitoses/10 HPF), NEC (poorly-differentiated, Ki-67 >20%, >20 mitoses/10 HPF) and MiNEN (mixed neuroendocrine-non-neuroendocrine neoplasm). Lung NEN was classified as typical carcinoid (<2 mitoses/10 HPF, no necrosis), atypical carcinoid (2-10 mitoses/10 HPF, focal necrosis) and NEC (>10 mitoses/10 HPF, extensive necrosis). Abdominal NENs other than GEP NENs were classified according to GEP NEN classification, and thymus NENs were classified according to the lung NEN classification. 

 Among all SRS examinations, 7 examinations identified 2 organs as the NEN primary sites; however, one site with high malignant grade according to the NEN classification or widespread development was represented. Multiple endocrine neoplasia type 1 (MEN 1) was diagnosed in 9 examinations, and functional NEN was diagnosed in 11 examinations (high gastrin levels=10, high vasoactive intestinal poly-peptide=1).

 CT examination was performed approximately three months before and after SRS examination in all 125 patients, and the CT diagnostic results were compared with SRS results. FDG-PET/CT was performed in 33 patients. Furthermore, SRS performed pre-resection was compared with CT and FDG-PET/CT performed pre-resection.

***SRS examination protocol***

 In-111 pentetreotide (OctreoScan, Fujifilm Toyama Chemical Co., Ltd., Tokyo, Japan) was intravenously administered at 111 MBq, and whole-body planar imaging and single-photon emission CT (SPECT) were performed at 4 and 24 h after administration (6). Furthermore, SPECT/CT was performed 24 h after administration. Imaging after 48 h was not performed at our hospital.

 For SRS examinations, Infinia Hawkeye4 (GE Healthcare, Milwaukee, Wisconsin) was used as a SPECT/CT system to collect images. Xeleris3.0 (GE Healthcare) was used as a workstation for image processing. SRS images were acquired using a large-field-of-view gamma camera fitted with a medium-energy collimator. Symmetrical 20% energy windows were centered over both photopeaks of 171 keV and 245 keV and the data from both windows were added. Whole-body images were acquired with 8 cm/min and spot images take 5-6 min. SPECT imaging of the appropriate regions was taken by 5 degrees sampling/rotation with 20-30 sec/step using 128×128 matrix. Diagnostic images were created by the radiological technologists on the workstation. Basically, it was the default setting based on the strongest accumulation part. Based on these images provided, it was diagnosed by a diagnostic radiologist.

 Although the use of laxatives is recommended for improving the detection of gastrointestinal lesions (7), our hospital does not follow this practice. Because inhibition of binding to the SSTR may occur during treatment with SSA, waiting for 4–6 weeks after the SSA use before SRS is performed is recommended (7). At our hospital, patients were instructed to wait at least 2 weeks; however, it was performed even within 2 weeks at the physician’s discretion.

***SRS Assessments***

 SRS uptake was not quantifiable and was therefore assessed using the Krenning scale (8, 9). The Krenning scale evaluates the degree of accumulation of lesions on a 5-point scale as Grade 0–4. The scales are classified as follows: Grade 0, no accumulation; Grade 1, less accumulation than the background liver; Grade 2, accumulation equivalent to the background liver; Grade 3, accumulation exceeding the background liver; and Grade 4, more accumulation than the background liver and equivalent to the spleen. Grades of ≥2 were assessed as cumulative positive. Because the evaluation of each NEN lesion in comparison with CT was difficult, those containing at least one lesion with grade of ≥2 on the Krenning scale were considered NEN-positive in the SRS examination.

 The NEN-positive rate in SRS examinations with NEN lesions was evaluated based on NEN histopathological classification. A total of 34 patients received SSA before the SRS examination, and the NEN-positive rate in SRS was similarly evaluated by NEN histopathological classification.

 For the comparison of superiority in detectability between SRS and CT, the following parameters were considered: CT only: when NEN lesions were detected by CT alone; SRS < CT: when one or more NEN lesions are detected by both, but there are lesion-existing organs detected by CT but not detected by SRS; SRS=CT: when one or more NEN lesions are detected by both, and lesion-existing organs are the same; SRS > CT: when one or more NEN lesions are detected by both, but there are lesion-existing organs detected by SRS but not detected by CT; and SRS only: when NEN lesions were detected by SRS alone, which were confirmed by gastrointestinal endoscopy or MRI rather than CT. The superiority of SRS to FDG-PET/CT in terms of detectability was similarly evaluated.

 For the detection of lesions by organ, NEN lesions were considered positive when positive accumulation was observed in at least one lesion in the organ. The positive ratio of NEN lesions by organ in SRS to the target lesions by organ in the comprehensive evaluation was evaluated by NEN histopathological classification.

 Furthermore, NEN lesions recognized only in SRS and non-NEN accumulation in SRS (3, 10) were mentioned.

## Results

 At least one NEN lesion was detected in 73% 

(91/125) in SRS with a positive Krenning score of grades ≥2. Meanwhile, in 3 examinations wherein target lesions were not diagnosed as NEN and 2 examinations in which the diagnosis was not confirmed, the Krenning score was 0 and undetectable in SRS.

**Figure 2:**
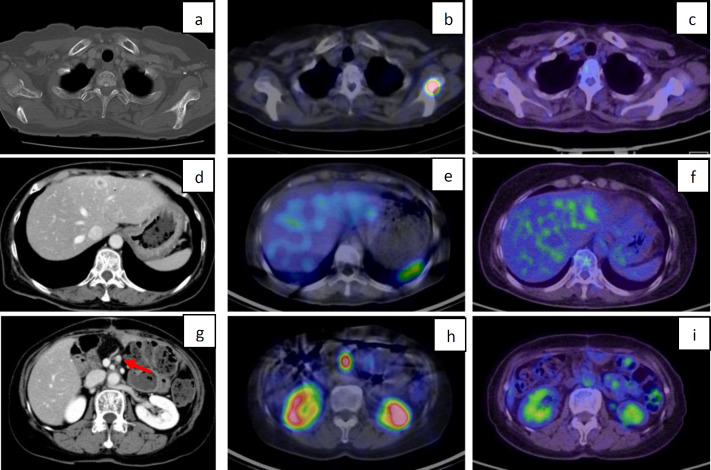
A female patient in her 70s was being treated with somatostatin analog for liver metastases and lymph node metastases after resection of pancreatic NEN (NET-G2)

**Figure 3:**
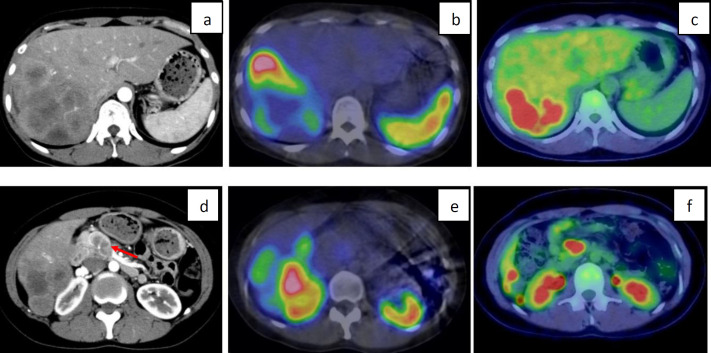
A female patient in her 30s with pancreatic NEC accompanied by liver metastases was undergoing chemotherapy.

 The detection rate by primary lesion site and NEN histopathological classification is shown in Table 2. In abdominal NEN (gastrointestinal tract, 38; pancreas, 62; and others, 14), the incidence was as follows: NET-G1=89% (49/55), NET-G2=78% (32/41) (Figure 2), NET-G3=66% (2/3), NEC=31% (4/13) (Figure 3), MiNEN=100% (1/1), and non-classified (NC)=0% (0/1). In thoracic NEN (lung, 9 and thymus, 2), the incidence was as follows: typical carcinoid=33% (2/6) and atypical carcinoid= 40% (2/5).

**Table2:** Detection rate of NEN lesions in 125 SRS examinations by primary site and histopathological classification of NEN

**NEN primary site**	**Abdomen**						**Chest**	
	**NET-G1**	**NET-G2**	**NET-G3**	**NEC**	**MiNEN**	**NC**	**Typical CT**	**Atypical CT**
**Stomach**		100％（1/1）		0％（0/1）	100％（1/1）			
**Duodenum**	95％（12/13）	75％（3/4）						
**Jejunum**	100％（1/1）							
**Colon**				0％（0/1）				
**Rectum**	88％（7/8）	50％（3/6）		50％（1/2）				
**Pancreas**	85％（22/26）	85％（23/27）	67％（2/3）	60％（3/5）		0％（0/1）		
**Liver**	100％（3/3）			0％（0/2）				
**Gall bladder**				0％（0/1）				
**Seminal vesicle**		100％（1/1）						
**Prostate**				0％（0/1）				
**Ovary**	100％（1/1）							
**Abdominal LN**	100％（3/3）	50％（1/2）						
**Lung**							33％（2/6）	0％（0/3）
**Thymus**								100％（2/2）
**Gastrointestinal tract**	91％（20/22）	64％（7/11）		25％（1/4）	100％（1/1）			
**Pancreas**	85％（22/26）	85％（23/27）	67％（2/3）	60％（3/5）		0％（0/1）		
**other**	100％（7/7）	67％（2/3）		0％（0/4）				
**Abdomen**	89％（49/55）	78％（32/41）	66％（2/3）	31％（4/13）	100％（1/1）	0％（0/1）		
**Chest**							33％（2/6）	40％（2/5）

NEN: neuroendocrine neoplasm

SRS: somatostatin receptor scintigraphy

NET-G: neuroendocrine tumor-grade

NEC: neuroendocrine carcinoma

MiNEN: mixed neuroendocrine-non-neuroendocrine neoplasm

NC: non-classified

CT: carcinoid tumor

LN: lymph node

 The incidence of lesions by organ site (total=226 organs) was 76% (58/76) for hepatic lesions, 61% (31/51) for pancreatic lesions, 77% (27/35) for lymph node lesions, 83% (20/24) for bone lesions, 82% (9/11) for duodenal lesions, and 41% (11/27) for other lesions(Figure 2). Table 3 shows the detection rate by lesion organ site and NEN histopathological classification.

**Table 3:** Detection rate of 226 organ lesions by organ with NEN lesions and histopathological classification of NEN in 125 SRS examinations

**Organ with NEN lesions**	**"Number of organs affected**	**Abdomen**						**Chest**	
		**NET-G1**	**NET-G2**	**NET-G3**	**NEC**	**MiNEN**	**NC**	**Typical CT**	**Atypical CT**
**Liver**	76	93％（28/30）	77％（23/30）	50％（1/2）	36％（4/11）	100％（1/1）			50％（1/2）
**Pancreas**	51	81％（21/26）	54％（7/13）	33％（1/3）	33％（2/6）		0％（0/1）	0％（0/2）	
**Lymph node**	35	100％（11/11）*	69％（11/16）		50％（2/4）	100％（1/1）		0％（0/2）	100％（2/2）
**Bone**	24	100％（6/6）*	100％（12/12）*	0％（0/1）	20％（1/5）			50％（1/2）	
**Duodenum**	11	90％（9/10）*	0％（0/1）						
**Lung**	8	100％（1/1）			0％（0/2）			0％（0/1）	25％（1/4）
**Rectum**	6	0％（0/1）	33％（1/3）		0％（0/2）				
**Peritoneum**	3	100％（1/1）			50％（1/2）				
**Brain**	2				0％（0/1）			0％（0/1）	
**Thymus**	2							100％（2/2）	
**Stomach**	1					100％（1/1）			
**Gall bladder**	1				0％（0/1）				
**Spleen**	1		100％（1/1）						
**Kidney**	1							0％（0/1）	
**Adrenal gland**	1								
**Urinary bladder**	1		0％（0/1）						
**Thyroid**	1		100％（1/1）*						
**Myocardium**	1	100％（1/1）*							50％（1/2）

NEN: neuroendocrine neoplasm

SRS: somatostatin receptor scintigraphy

NET-G: neuroendocrine tumor-grade

NEC: neuroendocrine carcinoma

MiNEN: mixed neuroendocrine-non-neuroendocrine neoplasm

NC: non-classified

CT: carcinoid tumor

*Included 15 organ lesions detected by SRS, not CT; NET-G1 bone lesions (n = 3), NET-G2 bone lesions (n = 8), NET-G1 lymph node lesions (n = 1), NET-G1 duodenal lesion (n = 1), NET-G2 thyroid lesion (n = 1), and NET-G1 myocardial lesion (n = 1)

 A comparison of the detectability of SRS and CT for NEN diagnosis is shown in Table 4-a. Sixty-four percent (80/125) of patients in SRS were evaluated to be equal to or better than CT in terms of detectability of NEN.

 NEN lesions detected only by SRS were found in 15 organs in 14 examinations, of which bone lesions were confirmed in 11 examinations, and lymph node, thyroid, myocardium, and duodenal lesions were confirmed in 1 examination each. In the case of myocardial and duodenal lesions, non-contrast-enhanced CT was performed due to contraindications to the use of contrast media.

 The evaluation of the superior detectability of SRS in 33 examinations that could be compared with FDG-PET/CT is shown in Table 4-b. Fifty-two percent (17/33) of SRS were evaluated to be equal to or better than FDG-PET/CT in the detectability of NEN. Among them, it was 90% (9/10) in NET-G1 cases and 73% (8/11) in NET-G2 cases. By contrast, NET-G3, NEC, and atypical carcinoid were predominantly diagnosed by FDG-PET/CT in 9 cases. Even in the same organ, SRS and FDG-PET/CT showed different degrees of accumulation and complementary distri-butions depending on the lesion (Figure 3). 

**Table 4-a:** Assessment of superiority of SRS and CT in the detectability of NEN (n = 125)

	**Abdomen**						**Chest**		**Total**
	**NET-G1**	**NET-G2**	**NET-G3**	**NEC**	**MiNEN**	**NC**	**Typical CT**	**Atypical CT**	
**CT only**	5	9	1	7		1	3	2	28
**SRS ** **＜** ** CT**	4	6	1	2				2	15
**SRS = CT**	41	16	1	4	1		2	1	66
**SRS ** **＞** ** CT**	4	9							13
**SRS only**	1								1
**Neither detected**	1	1							2
**Overall**	56	41	3	13	1	1	5	5	125

**Table 4-b:** Assessment of superiority of SRS and FDG-PET/CT in the detectability of NEN (n = 33)

	**Abdomen**						**Chest**		**Total**
	**NET-G1**	**NET-G2**	**NET-G3**	**NEC**	**MiNEN**	**NC**	**Typical CT**	**Atypical CT**	
**PET only**		1		1			1	2	5
**SRS < PET**	1		1	2				3	7
**SRS = PET**	5	2					1		8
**SRS > PET**	1	3							4
**SRS only**	3	3							6
**Neither detected**		2					1		3
**overall**	10	11	1	3	0	0	3	5	33

SRS: somatostatin receptor scintigraphy

CT: computed tomography

NEN: neuroendocrine neoplasm

NET-G: neuroendocrine tumor-grade

NEC: neuroendocrine carcinoma

MiNEN: mixed neuroendocrine-non-neuroendocrine neoplasm

NC: non-classified

CT: carcinoid tumor

FDG-PET: fluorine-18 deoxyglucose positron emission tomography

PET: positron emission tomography

 Among the 34 patients who used SSA medication before the SRS examinations, the median time from the day of the last SSA dose to the day of SRS examination was 27 days (7–42 days). The detection rate by primary lesion site and NEN histopathological classification in 34 patients is shown in Table 5. NET-G1 was detected in 100% (21/21), NET-G2 in 83% (10/12), and NEC in 0% (0/1) of the cases.

**Figure 4:**
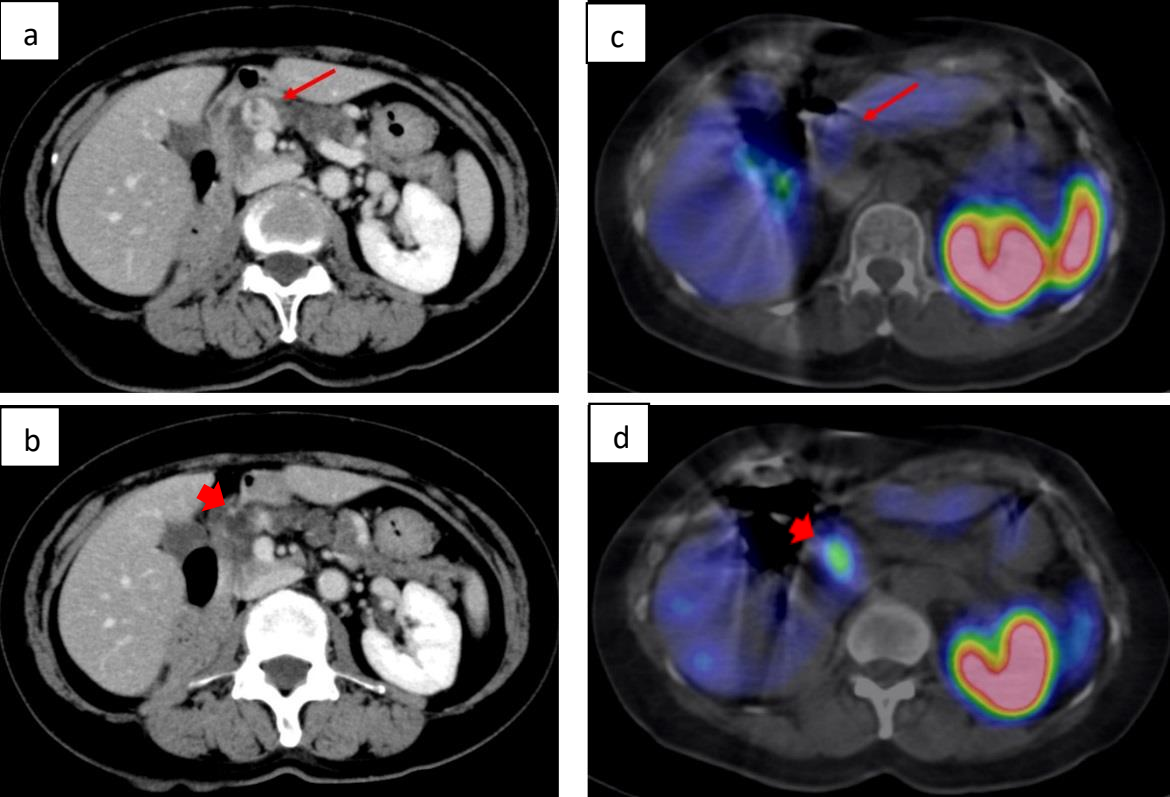
A female patient in her 60s showed a hypervascular mass lesion (**4a**. arrows) in the pancreatic head on contrast-enhanced CT, which occurred following right nephrectomy, but NEN was considered in the differential diagnosis, and SRS was performed. SRS-SPECT/CT (**4d**. arrowhead) showed increased accumulation in the uncus of the pancreas head, which was misidentified for accumulation in a mass lesion. Indeed, mass lesions do not accumulate in SRS (**4c**. arrow), and sites of SRS accumulation (**4d **arrowhead) are not visible on CT (**4b**. arrowhead). The resected tumor was diagnosed as pancreatic metastasis from renal cell carcinoma

 Overall, 15 significant accumulations other than NEN lesions were described in the SRS diagnostic report: 8 cases (6.4%) of pancreatic head hyperaccumulation (Figure 4), 4 cases of pituitary adenoma, 1 case of meningioma, 1 case of suspected thyroid adenoma, 1 case of suspected parathyroid hyperplasia, and 1 case of suspected adrenal adenoma.

**Table 5:** Detection rate of NEN lesions in 34 SRS examinations by primary site and histopathological classification of NEN in patients with SSA medication before SRS*

**NEN primary site**		**NET-G1**	**NET-G2**	**NET-G3**	**NEC**	**MiNEN**	**NC**
**Gastrointestinal tract**	n = 17	100％（12/12）	50％（2/4）		0%（0/1）		
**Pancreas**	n = 16	100％（8/8）	100％（8/8）				
**Ovary**	n = 1	100％（1/1）					
**overall**	n = 34	100％（21/21）	83％（10/12）		0%（0/1）		

NEN: neuroendocrine neoplasm

SRS: somatostatin receptor scintigraphy

SSA: somatostatin analog

NET-G: neuroendocrine tumor-grade

NEC: neuroendocrine carcinoma

MiNEN: mixed neuroendocrine-non-neuroendocrine neoplasm

NC: non-classified

CT: carcinoid tumor

* Median time to SRS examination, 27 days (7–42 days)

SRS-SPECT/CT (**2b**) shows a high-accumulation lesion in the left scapula that is not recognized by CT (**2a**) and FDG-PET/CT (**2c**), and the patient was diagnosed with bone metastasis.

Liver metastatic lesions observed on contrast-enhanced CT (**2d**) cannot be recognized on SRS-SPECT/CT (**2e**) and FDG-PET/CT (**2f**)

Abdominal lymph node metastasis is small on contrast-enhanced CT (2g arrow), whereas SRS-SPECT/CT (**2h**) shows high accumulation. Not detected by FDG-PET/CT (**2i**)

Pancreatic primary lesion (**3d**: contrast-enhanced CT arrow) does not show accumulation on SRS-SPECT/CT (**3e**), but shows high accumulation on FDG-PET/CT (3f arrow).

Liver metastases (**3a**, **d**: contrast-enhanced CT) show high accumulation on FDG-PET/CT (**3c**, **f**) as well as lesions with high accumulation on SRS-SPECT/CT (**3b**, **e**), showing a complementary accumulation distribution

## Discussion

 Somatostatin is a peptide hormone secreted from nerve or endocrine cells, which are distributed in the body and it inhibits the secretion of several secondary secretory hormones via SSTRs (11). Although somatostatin is activated by binding to SSTRs, these receptors are expressed at a high rate in various central nervous system tumors, including NEN (10). Particularly, the SSTR subtypes 2 and 5 are known to be highly expressed among the five subtypes of SSTR in NEN (12), and the development of a SSA that is stable for a longer time than somatostatin with a shorter half-life in blood is desired as a target for diagnosis and treatment of NEN. In-111 pentetreotide (OctreoScan) has been developed for diagnosis and can be imaged using a gamma camera (1, 2). Although the use of SRS has been prevalent in Europe and the United States for >20 years, in Japan, it was introduced in January 2016 for NEN diagnosis (3, 13).

 The significance of SRS examination in relation to NEN is clearly stated in the NEN clinical practice guidelines (10, 14, 15); however, NEN itself is a rare disease, and the frequency of SRS examination is not frequent. Therefore, the usefulness of SRS has not sufficiently been verified in Japan, and hence, consecutive cases conducted in our hospital were reviewed.

 Although the diagnostic ability of SRS reportedly varies depending on the degree of NEN differentiation, Binderup et al. have reported that the detection rate by SRS for NEN, including gastrointestinal, pancreatic, and lung primary lesions by Ki-67 index was 87% for Ki-67 index <2%, 96% for 2%–15%, and 69% for ≥15% (16). In other studies, the incidence of SSTR was lower in NET-G3 and NEC than in NET-G1 and NET-G2 (17-19). In the present study, the detection rate of NEN lesions in 125 SRS examinations that had target lesions as NEN was 89% for NET-G1, 78% for G2, 66% for G3, and 31% for NEC in the abdominal NEN. In the chest NEC, it had 33% for typical carcinoids and 40% for atypical carcinoids. Our findings are seen to be similar to those previously reported.

 Also, the detection rate of SRS by NEN lesion organ site was as follows: liver lesions, 76%; pancreatic lesions, 61%; lymph node lesions, 77%; bone lesions, 83%; and duodenal lesions, 82%. The detection rate of liver metastasis was inferior to 89% as reported by Scigliano et al. (20) but was higher than 52% as reported by Gagriel et al (21). The diagnosis of bone metastasis was considered to have led to an early detection of bone metastasis lesions, the detection of which using CT was difficult. However, because not all NENs are detected in SRS, it was considered that diagnosis and follow-up combined with CT, MRI, and other modalities are essential.

 Furthermore, Binderup et al. reported that the diagnostic rate of NEN in FDG-PET/CT was 41% for Ki-67 index <2%, 73% for 2%–15%, and 92% for ≥15% (16). In the present study, FDG-PET/CT was infrequently performed, but FDG-PET/CT showed diagnostic superiority in cases of NET-G3, NEC, and atypical carcinoid compared with SRS. The degree of accumulation of SRS and FDG-PET/CT differed or showed complementary accumulation distribution depending on the lesion even in the same organ, suggesting that the degree of differentiation and expression of SSTR within the lesion were heterogeneous (6, 22, 23).

 Chan et al. reported that NETPET grading by the combined evaluation of the SSTR PET and FDG-PET correlated with the prognosis of NEN (24). PET using Ga-68-labeled octreotide reportedly has good affinity for SSTRs and excellent sensitivity and spatial resolution (25, 26). Practically, SSTR-PET may be convenient in that it can be taken in one day than SRS with In-111 pentetreotide. However, it has not been currently introduced in Japan.

 If SSA is used as a treatment for NEN, it is recommended that SSA medication should be suspended 4–6 weeks before SRS examination because SSA prevents In-111 pentetreotide from binding to SSTR on target lesions (7). However, in the present study, among 34 patients who used SSA medication before SRS examination, the detection rate of NEN in SRS was high at 100% in NET-G1 and 83% in NET-G2. The binding of SSA therapeutics to SSTRs in normal organs may have reduced the physiological accumulation in SRS and rather improved the contrast with target lesions (27).

 Pancreatic polypeptide (PP) cells express SSTRs, of which, many are considered to be located in the pancreatic head. Particular attention should be given to SSTR accumulation in PP cells in SRS. In particular, patients with diabetes mellitus reportedly have a high frequency of accumulation in PP cells with SRS (9, 28). In the present study, although the correlation with diabetes mellitus was unknown, high point-like accumulation that was judged as physiological accumulation and not NEN lesions, along with other diagnostic imaging was observed in 6.4% of cases in the pancreatic uncus. It was less frequent than the previously reported 26%, but could be difficult to determine if there were lesions in the vicinity.

 It is assumed that SRS is not specific for NEN considering that there are other diseases or tissues that express SSTR other than NEN (10). The present study experienced the accumulation of known pituitary adenoma and meningioma, which was discovered following SRS (3). Accumulations suggestive of adrenal adenoma, thyroid adenoma, and parathyroid hyperplasia were also observed.

 The present study also has several limitations. First, this was a retrospective analysis that was performed at a single institution. Second, not all lesions were histopathologically evaluated. The histopathological classification of NEN comprised an evaluation of the site where the tissue sample was obtained, and it was designated as the representative NEN histopathological classification of the patient. Even in the same patient, there are differences in SRS and FDG-PET/CT accumulation depending on the lesion, and NEN histopathological classification may differ depending on the lesion site. The site of specimen collection should be selected with reference to SRS and FDG-PET/CT when predicting prognosis and selecting chemotherapy. In addition, since the timing of the pathological diagnosis and the SRS examination did not always coincide, the actual histopatho-logical classification at the time of the SRS test may have changed over time and the course of treatment. Third, SRS image display was evaluated based on the default image at the image drawing workstation; however, the image display was entrusted to the responsible radiological technologist. The delineation of the same lesion may have differed by image display. Owing to the difficulty of a quantitative assessment of SRS accumulation, there remains a possibility of image display bias. SPECT/CT was not obtained in the regions where the lesion was not recognized on the previous CT and the planar whole-body images in SRS. Therefore, evaluation in regions where SPECT/CT was not performed was insufficient. Fourth, in the comparison between SRS and FDG-PET/CT, FDG-PET/CT was not performed in all cases, so both were compared in cases where FDG-PET/CT was clinically required. It was necessary to consider the case bias in that respect. Although there were some limitations to this study and SRS alone could not evaluate the entire NEN, the usefulness of SRS in the investigation of NEN metastasis and recurrence diagnosis was high.

 Peptide receptor radionuclide therapy (PRRT), which has already been conducted overseas, is an internal radiation therapy that combines SSA with α-ray or β-ray emitting nuclide with SSTR of NEN lesion (29, 30). PRRT is expected to be implemented in Japan in the near future. Confirmation of SSTR expression is essential for PRRT implementation, which will increase the demand for SRS. Upon the introduction of PRRT in Japan, SRS will become an essential examination and indices for further quantification will be required. 

## Conclusion

 The present study demonstrated the detectability of SRS in clinical practice for NEN diagnosis and verified its usefulness.

## Conflict of Interest

 All authors have no conflicts of interest to disclose in this study.

## Ethical Statement

 This retrospective study was approved by the Institutional Review Committee of our institution. Written informed consent for all patients was waived, but an opportunity to opt out was provided.
